# Effects of different grains on bacterial diversity and enzyme activity associated with digestion of starch in the foal stomach

**DOI:** 10.1186/s12917-022-03510-2

**Published:** 2022-11-17

**Authors:** Xiao Bin Li, Xin Xin Huang, Qian Li, Xuan Yue Li, Jia Hao Li, Chao Li, Lin Jiao He, Hong Xin Jing, Kai Lun Yang

**Affiliations:** College of Animal Science, Xinjiang Agricultural University, Xinjiang Key Laboratory of Herbivore Nutrition for Meat & Milk Production, Urumqi, Xinjiang, 830052 China

**Keywords:** Grains, Foal, Stomach, Bacteria, Starch-digesting enzymes

## Abstract

**Background:**

Compared with the stomach of ruminant cattle, the stomach of horse is small and mainly for chemical digestion, but the microorganisms in the stomach play an important role in maintaining the homeostasis of the internal environment. Due to the complexity of the microbes in the stomach, little is known about the diversity and structure of bacteria in the equine stomach. Grains are the main energy source for plant-eating livestock and energy is derived through enzymatic hydrolysis of grains into glucose or their microbial fermentation into Volatile fatty acids (VFA). However, the mechanism through which these ingested grains are chemically digested as well as the effect of these grains on the stomach remains elusive. This study explored the effects of feeding different grains (corn, oats, and barley) on bacterial diversity, structure, and composition in the foal’s stomach content. Furthermore, the effects of different grains on the vitality of starch digestion-related stomach enzymes were investigated.

**Results:**

No significant differences were observed (*P* > 0.05) in the bacterial rarefaction curves of Operational Taxonomic Units (OTUs) and diversity of the stomach microbiota in all foals. This study also revealed the statistical differences for Firmicutes, Cyanobacteria, Actinobacteria, Fibrobacteres, Lactobacillaceae, Streptococcaceae, Unidentified_Clostridiales, Prevotellaceae, *Lactobacillus, Streptococcus, Unidentified_Cyanobacteria, Unidentified_Clostridiales, Lactococcus, Sphingomonas, Lactobacillus_hayakitensis, Lactobacillus_equigenerosi, and Clostridium_perfringens*. The linear discriminant analysis effect size analysis revealed 9 bacteria at each classification level. The functional analysis of species information by using FAPROTAX software was able to predict 35 functions, and the top 5 functions were chemoheterotrophy, fermentation, animal_parasites_or_symbionts, nitrate_reduction, and aerobic_chemoheterotrophy. The study also revealed statistical differences for pH, glucose concentration, β-amylase, maltase, and amylase.

**Conclusions:**

The different grains had no significant effect on the microbial diversity of the stomach content of the foal. However, the relative bacterial abundances differed significantly in response to different diets. Particularly, oats fed to the foals significantly increased the relative abundance of Firmicutes, Lactobacillaceae, *Lactobacillus,* and *Lactobacillus_hayakitensis*. The grain had no significant effect on the pH of the stomach content, glucose concentration, and enzyme viability in the foal.

## Introduction

Food digestion in animals comprise an interplay of various physical, chemical, as well as microbial processes. The gastrointestinal tract is among the vital organs of the body, mainly involved in digesting and absorbing nutrients in animals. The structure and function of the gastrointestinal tract are closely related to the growth performance and health status of the animals. The gastrointestinal tract is inhabited by numerous microorganisms. These microorganisms participate in various physiological and biochemical functions of the host by establishing a micro-ecological balance between interdependence and interaction between the host and microorganisms. Significant variations in the microbial species, numbers, and functions were noted between the different intestinal segments. For example, the microorganisms in the intestinal lumen, intestinal mucosa, intestinal wall, and chyme vary even if they are from the same part of the intestine [1, 2].

Horses are monogastric herbivores with their stomach mainly responsible for chemical digestion; however, microorganisms in their stomach are crucial for maintaining the digestive physiology of the organ. Varloud et al. demonstrated that bacteria such as *Streptococcus bovis, S. equi (S. equinus), Lactobacillus salivarius, L. mucosae (L. mucosae), L. delbrueckii (L. delbrueckii)*, and *Mitsuokella jalaludinii* can use starch or nonstructural carbohydrates in the diet as a carbon source [3]. In addition, microbial changes in the stomach can affect the health of horses. Coenen et al. identified that changes in certain stomach microorganisms are directly responsible for hoof laminitis [4].

Grains in the horse’s diet mainly increase energy intake [5]. Horses mainly feed on corn, oats, and barley, which comprise similar amounts of amylose and amylopectin, but variable proportions of starch polysaccharides and starch granules of various sizes. This leads to the variation in the utilization of different digestive nutrients, thereby affecting the gastrointestinal tract of the horse [6, 7]. Differences have been observed in the digestibility of grain granules in the stomach and small intestines of horses [8]. The undigested starch in the stomach and small intestine reaches the hindgut through the small intestine to undergo microbial fermentation and degradation. Starch fermentation increases the number of amylolytic bacteria, including lactobacilli and streptococci, which increases the concentration of lactic acid and short-chain fatty acids, thereby lowering the hindgut pH, maintaining the hindgut health, and changing the types and abundances of intestinal microbes [9]. However, to date, no study has elucidated the mechanism through which different grains affect the microbial composition of the horse’s stomach. Therefore, this study investigated the effects of supplemental feeding of different grains (corn, oats, and barley) on bacterial diversity, structure, and composition in the stomach content of the weaned foals, as well as the effects of different grains on the activity of enzymes associated with starch digestion in the stomach.

## Materials and methods

### Animals

The Kazakh horse, a species of local breed in China, was selected in this study [10]. This study included 18 healthy 5-month-old weaned foals of the Kazakh toad breed with a starting bodyweight of 112.4 ± 7.5 kg. The foals were born in March and weaned in August, and the trial was conducted between August and October (60 days). The foals were selected from the herds occupying the same local pasture, were found to be clinically normal upon inspection, and showed no history of systemic illness. Before the foals were weaned, they were dewormed with ivermectin. The foals were randomly allocated to the corn, oats, or barley group based on either of the three diets, and each group comprised 6 foals.

### Feed

The foals were fed with a diet having forage and concentrate. The forage comprised alfalfa hay and hay, fed as a mixture at a 2:1 ratio, and could be consumed to yield 1.5 kg of dry matter (DM) for each 100 kg of body weight. The amounts of starch fed were set at 2 g starch (DM)/kg body weight [11]. The amount of additional starch required was 224 g/day for the mean weight of the foal between birth and 30 days being 112 kg. The average weight of the foal from 31 to 60 days of age was 126 kg, and starch was added at 252 g/day. The actual grain supplement was calculated, and an equal amount of the concentrate supplement was added (Table 1). The foals gradually adapted to dietary starch within 10 days of the pre-feeding period. The dietary grains in each test group were steam-flaked and puffed under the same processing conditions, and the nutrient levels of the foal diet during the test period are shown in Table 2.

**Table 1:** Composition of concentrate supplement(%)

The raw material	Corn group	Oats group	Barley group
Steam-pressed corn	60	–	–
Steam-pressed oats	–	66	–
Steam-pressed barley	–	–	66
Soybean meal	36	30	30
Calcium hydrogen phosphate	2	2	2
Limestone	1	1	1
Premix^a^	0.5	0.5	0.5
Salt	0.5	0.5	0.5

^a^The premix provided the following per kilogram of concentrate supplement: Vitamin A 3000 IU, Vitamin B_1_ 20 mg, Vitamin B_2_ 20 mg, Vitamin B_6_ 6 mg, Vitamin C 20 mg, Vitamin D 1000 IU, Vitamin E 500 IU, Pantothenic acid 10 mg, Nicotinamide 100 mg, Cu 25 mg, Fe 107 mg, Mn 81 mg, Zn 74 mg, I 6 mg, Se 14 mg, Co 3 mg, Choline chloride 120 mg

**Table 2:** Nutrient levels of hay, alfalfa, and concentrate supplements (Dry Matter, DM basis)

Nutrient	Hay	Alfalfa	Corn group	Oats group	Barley group
Dry Matter,DM (%)	89.75	87.56	92.44	93.28	91.93
Organic Matter,OM (%)	91.80	92.21	94.46	93.32	93.90
Crude Protein,CP (%)	10.75	20.56	26.26	26.09	26.86
Gross Energy,GE (MJ/kg)	18.93	19.53	18.25	18.29	18.26
Starch (%)	3.63	4.46	37.68	37.00	38.27
Neutral Detergent Fiber,NDF (%)	60.00	52.60	24.46	27.04	24.79
Acid Detergent Fiber,ADF (%)	41.82	45.21	5.10	9.09	5.71
Calcium,Ca (%)	0.33	1.50	1.06	1.45	1.14
Phosphorus,P (%)	0.27	0.25	0.94	1.02	1.02

### Management

The foals were individually housed in partially covered confinements of red brick (size: 8 × 6 m) for 60 days of the study. Each unit comprised an automatic water source, and the feeding area was equipped with a red brick floor and a large wooden tub secured to the wall. After the foals finished feeding on the daily grain, they were driven from the unit to the outdoor activity field for free movement and frolic.

### Gastric tube sampling

On the 58th day of the trial, the stomach content was collected from the foals by using an oral gastric tube. On that day, the foals were housed in the pens, such that the foal’s heads were held in place by the four keepers, and the foal’s tongues were pressed down by using an opener to open their mouths. The foals tolerated the passing the stomach tube through the mouth without using a twitch. The stainless steel cartridge of the sampling tube was lubricated by the veterinarian, and the snake tube was sprung at one end of the gastric tube (Gastric tube MDW15; Chengdu Huazhi Kaiwu Technology Co., Ltd., Chengdu, China) such that it could enter slowly through the mouth of the foal, with the end of the tube reaching the pharynx until resistance could be felt. Caution was exercised in not forcefully pushing, pumping the cartridge, and springing the snake tube back and forth to produce a swallowing action in the foal and to send the cartridge and spring the snake tube into the esophagus in a smooth motion. Depending on the foal size, the gastric tube would be able to enter up to a depth of 90–102 cm and the stomach content could be aspirated using a syringe. The horse was then aspirated to determine whether the gastric tube had successfully entered the stomach. The sampling tube was inserted into the stomach and allowed to remain inside for approximately 5–10 min to enable the stainless steel cartridge to sink into the stomach content. First, air was expelled from the stomach tube by using a syringe. After the air was extracted with the syringe, the latex tube was pinched or folded to prevent it from leaking, and then the air was expelled from the syringe. Subsequently, the stomach content was extracted using a sterile 200 mL syringe connected to the latex gastric tube, frozen, and stored at − 80 °C. After collecting the sample of the stomach content, the gastric tube was slowly removed.

### Microbial DNA extraction from stomach content samples

Total genomic DNA from samples was extracted using the CTAB/SDS method [12]. DNA concentration and purity was assessed on 1% agarose gels. DNA was diluted to 1 ng/μL by using sterile water, The DNA was handed over to Novogene for processing (Novogene, Beijing, China).

### V3-V4 16S rRNA gene amplification and sequencing

The universal primers 341F 5′-CCTAYGGGRBGCASCAG-3′ and 806R 5′-GGACTACNNGGGTATCTAAT-3′ [13]. were used to obtain the PCR amplicons for paired-end sequencing on an Illumina MiSeq platform at Novogene (China).

16S rRNA genes were amplified using the specific primer with the barcode. All PCR reactions were carried out in a 30-μL reaction mixture with 15 μL of Phusion® High-Fidelity PCR Master Mix (New England Biolabs); 0.2 μM of forward and reverse primers, and approximately 10 ng template DNA. Thermal cycling consisted of initial denaturation at 98 °C for 1 min, followed by 30 cycles of denaturation at 98 °C for 10 s, annealing at 50 °C for 30 s, elongation at 72 °C for 30 s, and extension at 72 °C for 5 min [12, 14, 15].

Sequencing libraries were generated using the Illumina TruSeq DNA PCR-Free Library Preparation Kit (Illumina, USA) following manufacturer’s recommendations, and index codes were added. The library quality was assessed using the Qubit@ 2.0 Fluorometer (Thermo Scientific) and Agilent Bioanalyzer 2100 system. Finally, the library was sequenced on an Illumina NovaSeq platform, and 250-bp paired-end reads were generated.

### Bioinformatics analyses

Paired-end reads were assigned to samples based on their unique barcodes and truncated by cutting off the barcodes and primer sequences. Then, the paired-end reads were merged using FLASH (Version 1.2.7)^1^ [12]. Quality filtering of the raw tags were performed under specific filtering conditions to obtain high-quality clean tags [16] according to the QIIME (Version 1.9.1)^2^ [14] quality-controlled process. Next, the tags were compared with the reference database (Silva database)^3^ using the UCHIME algorithm (UCHIME algorithm)^4^ [17] to detect chimera sequences, which were then removed [18]. Then, the effective tags were finally obtained.

Sequences analysis was performed using Uparse software (Uparse Version 7.0.1001)^5^ [19]. Sequences with ≥97% similarity were assigned to the same operational taxonomic units (OTUs). Representative sequence for each OTU was screened for further annotation. For each representative sequence, the Silva database^6^ [20] was used based on Mothur algorithm to annotate taxonomic information. To study the phylogenetic relationship of different OTUs and the difference in the dominant species in different samples (groups), multiple sequence alignment was performed using the MUSCLE software (Version 3.8.31)^7^ [21]. OTU abundance information was normalized using a standard of sequence number corresponding to the sample with the least sequences. Subsequent analyses of alpha diversity and beta diversity were all performed based on these output normalized data.

Alpha diversity is applied for analyzing the complexity of species diversity for a sample through 6 indices, namely Observed-species, Chao1, Shannon, Simpson, ACE, and Good’s -coverage. All these indices in our samples were calculated using QIIME (Version 1.9.1) and displayed with R software (Version 2.15.3).

Beta diversity analysis was performed to evaluate differences in samples in species complexity. Beta diversity on both weighted and unweighted unifrac were calculated using QIIME software (Version 1.9.1). Cluster analysis was preceded by principal component analysis (PCA), which was applied to reduce the dimension of original variables using the FactoMineR package and ggplot2 package in R software (Version 2.15.3). Principal coordinate analysis (PCoA) was performed to obtain principal coordinates and visualize complex, multidimensional data. A distance matrix of weighted or unweighted unifrac among samples obtained before was transformed to a new set of orthogonal axes, by which the maximum variation factor is demonstrated by first principal coordinate, and the second maximum one by the second principal coordinate, and so on. The PCoA analysis was performed using the WGCNA package, stat packages, and ggplot2 package in R software (Version 2.15.3). Linear discriminant analysis (LDA) effect size (LEfSe) analysis was performed using LEfSe software with a default screening value of 4 for the LDA score. Metastats analysis was performed using R software at each classification level (phylum, class, order, family, genus, and species) by conducting the permutation test between groups to obtain *p* values, and then, the Benjamini and Hochberg false discovery rate method was used to correct for p values to obtain q values [21]. Anosim, MRPP, and Adonis analyses were performed using the Anosim function, MRPP function, and Adonis function of the R vegan package, respectively. AMOVA analyses were performed using the amova function of Mothur software. Species analysis for significant differences between groups was performed using the R software for the between-group t test and the results were plotted.

Tax4Fun function prediction was conducted using the nearest neighbor method based on minimum 16S rRNA sequence similarity by extracting prokaryotic whole-genome 16S rRNA gene sequences from the KEGG database and comparing them to the SILVA SSU Ref NR database (BLAST bitscore > 1500) by using the BLASTN algorithm to create a the functional information of the KEGG prokaryotic whole genome annotated by both UProC and PAUDA methods was corresponded to the SILVA database, and the SILVA database functional annotation was realized. The sequenced samples were clustered into OTUs using the SILVA database sequences as reference sequences to obtain functional annotation information.

### Determination of pH and glucose concentration

The pH of the aspirated stomach contents was determined using a calibrated portable pH meter (FiveEasy22-Meter, Mettler-Toledo International Trading (Shanghai) Co., China) having an accuracy of 0.01. The stomach content was homogenized and filtered through four layers of gauze. Then, the amount of glucose in the stomach content was determined using a glucose kit (glucose oxidase method, A154–1-1; Nanjing Jiancheng Bioengineering Institute, Nanjing, China).

### Determination of the activity of starch-digesting enzyme

A commercial kit (colorimetric method, A082–3-1; Nanjing Jiancheng Bioengineering Institute, Nanjing, China) was used for determining the maltase activity of the stomach content. The stomach content sample was homogenized and filtered through four layers of gauze. Then, 1 mL of the filtered stomach content sample was mixed with 9 mL of saline placed in an ice water bath and centrifuged at 1500×*g* for 10 min. Later, 10 μL of the supernatant was collected and mixed with the reaction solution in the test kit. The mixture was allowed to react at 37 °C for 15 min in a water bath. The reaction was terminated, and the OD value was measured using a visible spectrophotometer at 505 nm and 1-cm optical diameter. Then, maltase activity was calculated. One unit maltase activity was defined as 1 nmol of maltose per minute of hydrolysis per milligram of protein tissue at 37 °C and pH 6.0.

The β-amylase activity of the stomach content was determined using a commercial kit for β-amylase (colorimetric method, C016–2-1; Nanjing Jiancheng Bioengineering Institute, China). The stomach content sample was homogenized and filtered through four layers of gauze. Then, 0.1 mL of the filtered stomach content sample was mixed with 1 mL of distilled water at 20 °C–25 °C. Later, the mixture was centrifuged at 3000×*g* for 10 min. The supernatant was then taken in a 10-mL volumetric flask. The volume was fixed with distilled water, and the mixture was shaken well to form the amylase stock solution. Approximately 1 mL of the amylase stock solution was aspirated, and 4 mL of distilled water was added to the solution. The mixture was shaken well to obtain an amylase dilution solution for determining the total activity of (α + β) amylase. The reaction was carried out at various temperatures according to the manufacturer’s instructions for the commercial kit, and the reaction mixture was then allowed to stand for 10 min at 20 °C–25 °C. The OD value was measured using a visible spectrophotometer at 540 nm, and the β-amylase activity was calculated. The units were defined according to the sample mass: 1 unit of β-amylase activity was defined as 1 mg of reducing sugar per gram of sample catalyzed per minute in the reaction system.

To determine the amylase, the iodine–starch colorimetric method was used. The stomach content sample was homogenized and filtered through four layers of gauze. Then, 1 mL of the filtered gastric fluid was mixed with 9 mL of saline and centrifuged at 1500×*g* for 10 min, followed by dilution of the supernatant with saline for testing. The samples were made to react with 0.4 mg/mL substrate buffer for 7.5 min at 37 °C in an accurate water bath. Subsequently, the iodine solution was prepared by diluting 0.1 mol/L iodine stock solution with double-distilled water at a 1:9 ratio. Colorimetric measurements were then performed using a visible spectrophotometer (MAPADA V-1800PC, Shanghai MAPADA Instruments Co. China) at a wavelength of 660 nm. The OD value was determined, and the AMS viability was calculated.

## Statistical analysis

### pH, glucose concentration, and enzyme viability analyses

The data obtained by assessing stomach content pH, glucose, and starch-digesting enzymes of horses were analyzed as a randomized complete design by using the general linear model procedure in SAS software (version 9.2, SAS Inst. Inc., Cary, NC, USA) [22], which is based on the statistical model: Y_ij_ = μ + T_i_ + e_ij_. Where Y_ij_ is observation (stomach content pH, glucose, and starch-digesting enzymes), μ is the general mean, T_i_ is the effect of sources of grains in the experimental diet, and e_ij_ is the standard error term.

## Results

### Rarefaction curve and OTUs

**Fig. 1:**
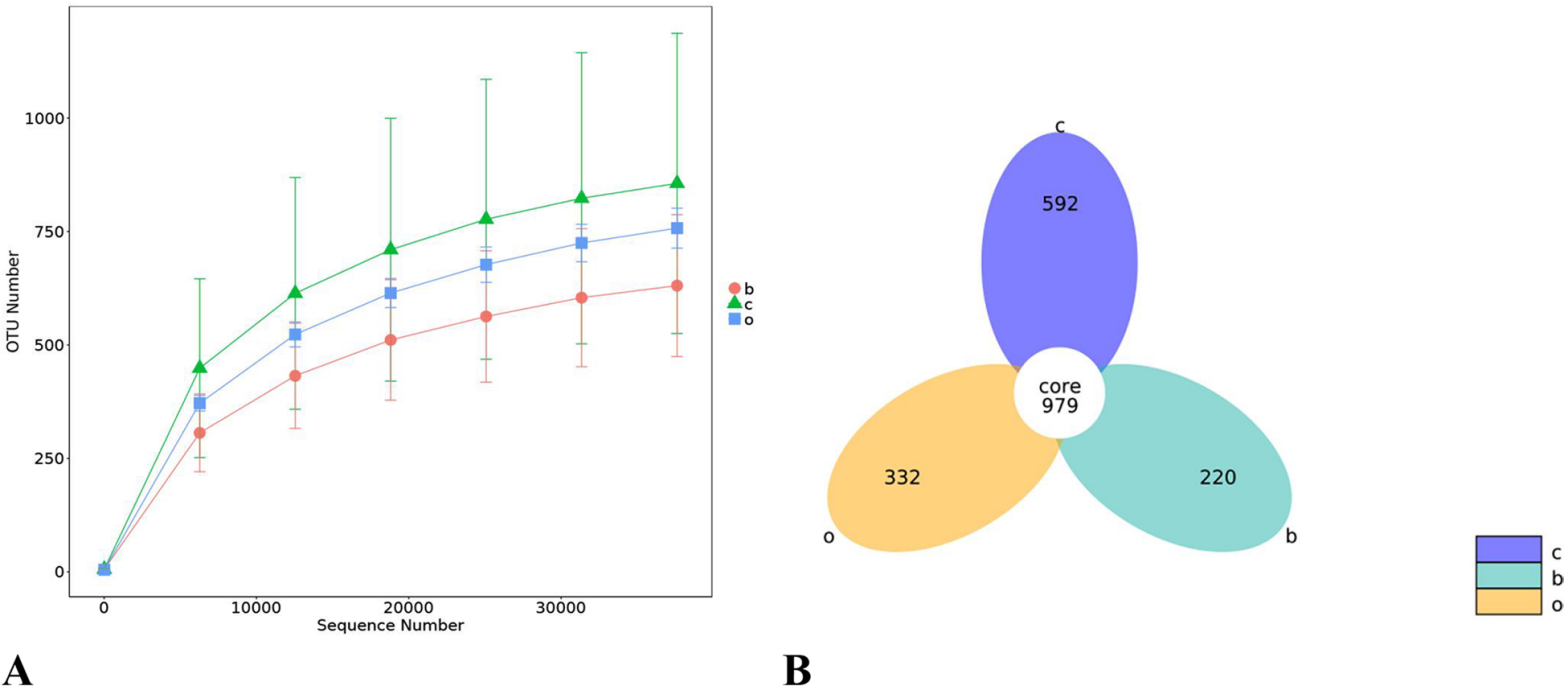
Effect of different grain sources on the bacterial population in the stomach content of foals. “**A**” The diagram represents the rarefaction curve of bacteria in the stomach content of foals. The groupings indicated by the letters in the figure are shown in the legend on the right, with the orange circle b representing the barley group, the green triangle c representing the corn group, and the blue square representing the oats group. “**B**” indicates the OTUs of bacteria in the stomach content of foals. The groupings indicated by the letters in the figure are shown in the legend on the right, with c and blue represent the number of all OTUs in the corn group, o and orange represent the number of all OTUs in the oats group, and b and green represent the number of all OTUs in the barley group

As observed from Fig. 1-A, the rising trend of the horizontal coordinate sequence number of the dilution curve of the three dietary groups was flat after 30,000, and the slope of the curve was smooth with small changes, indicating that the sequencing volume of each group had been sufficient. Moreover, the alpha diversity index of the samples would not change significantly, that is, the alpha diversity index of the samples reached stability.

Based on the results of OTUs (Fig. 1-B) obtained from clustering and the requirements for the research where each colored circle represented a group, the OTUs common to different groups and unique to each group were analyzed and plotted as a petal diagram using R software (Version 3.0.3, R package = VennDiagram). Moreover, the numbers in the overlapping part of the circles represented the number of OTUs shared among the groups, whereas those in the non-overlapping part represented the number of group-specific OTUs. This study included 979 OTUs common to the three groups, 592 unique to the corn group, 332 unique to the oats group, and 220 unique to the barley group. Moreover, the corn group had a greater variety of bacteria.

### Alpha diversity indices

Alpha diversity indices of the bacteria found in the stomach content [23] are presented in Table 3. The Chaol and ACE indices of the alpha diversity index were used to assess the abundance of the microflora. The greater the Shannon diversity index, the higher the diversity of the microbial community. No significant effect of the different grains was observed on the bacterial diversity in the foals’ stomach content.

**Table 3:** Effect of different sources of grains on the alpha diversity of the weaned foal’s stomach

	Corn group	Oats group	Barley group	SEM	*P-*Value
observed_species	797.60	693.83	575.50	87.37	0.2608
shannon	4.29	3.44	3.64	0.39	0.3466
simpson	0.75	0.73	0.79	0.04	0.5988
chao1	805.19	702.76	582.41	87.79	0.2614
ACE	823.04	721.09	598.57	89.05	0.2653
goods_coverage	1.00	1.00	1.00	0.00	0.3285
PD_whole_tree	52.98	51.43	42.76	5.28	0.3787

### Principal co-ordinate and principal component analyses

**Fig. 2:**
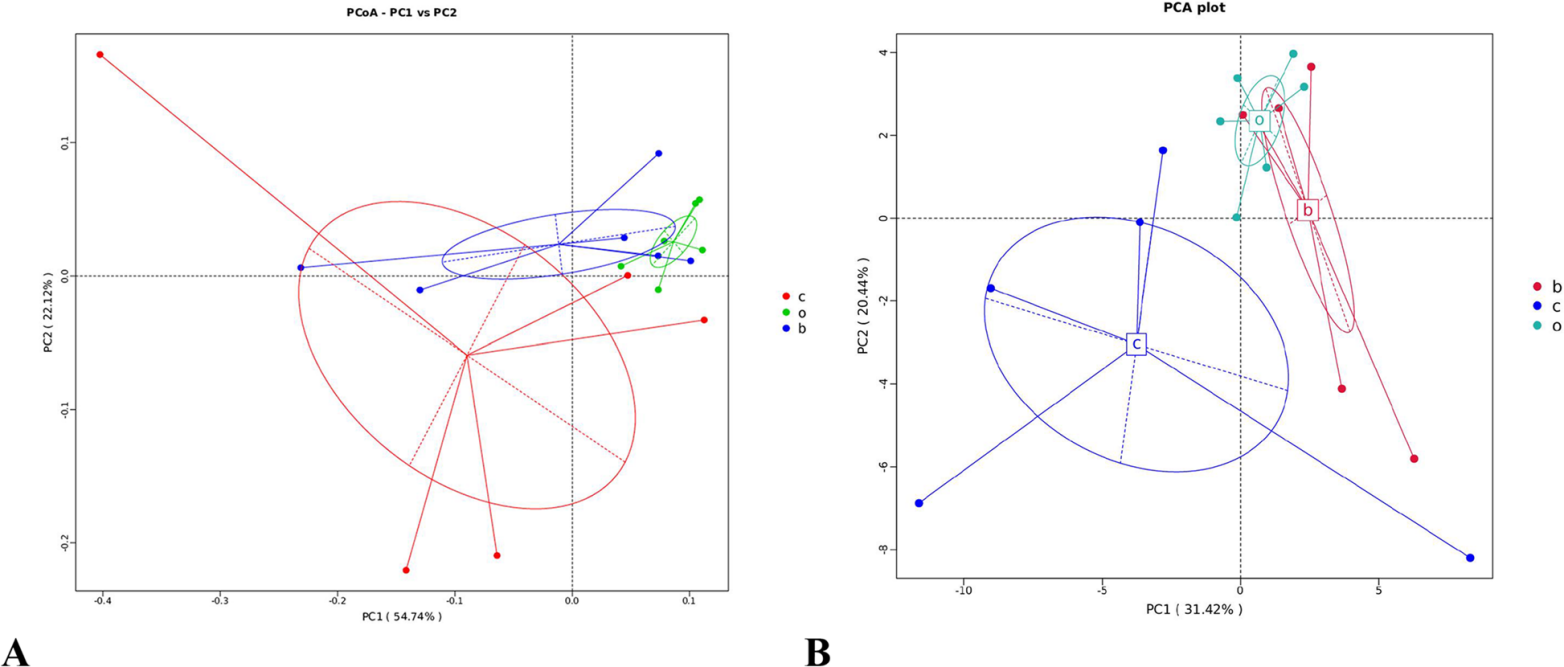
Effects of different grain sources on the bacterial PCoA (A) and PCA (B) in the stomach content of weaned foals. “**A**”PCoA diagram. The groupings indicated by the letters in the figure are shown in the legend on the right, red and c represent the corn group, green and o represents the oats group, and blue and b represents the barley group. “**B**”PCA diagram. The groupings indicated by the letters in the figure are shown in the legend on the right, red and b represents the barley group, blue and c represents the corn group, and green and o represents the oats group

Results of the PCoA (Principal Co-ordinates Analysis) analysis demonstrated the horizontal coordinates indicating one principal component, which contributed 54.74% to the sample variation, and the vertical coordinates indicating the other principal component, which contributed 22.12% to the sample variation. The similarity between bacteria in the oats and barley groups was higher as evident in Fig. 2-A.

The PCA (Principal Component Analysis) results were significant, with the horizontal coordinate indicating the first principal component, contributing 31.42% to the sample variance. The vertical coordinate, on the other hand, indicated the second principal component, contributing 20.44% to the sample variance. Thus, Fig. 2-B presents a higher similarity between the bacteria in the oats and barley groups.

### Relative bacterial abundance

**Fig. 3:**
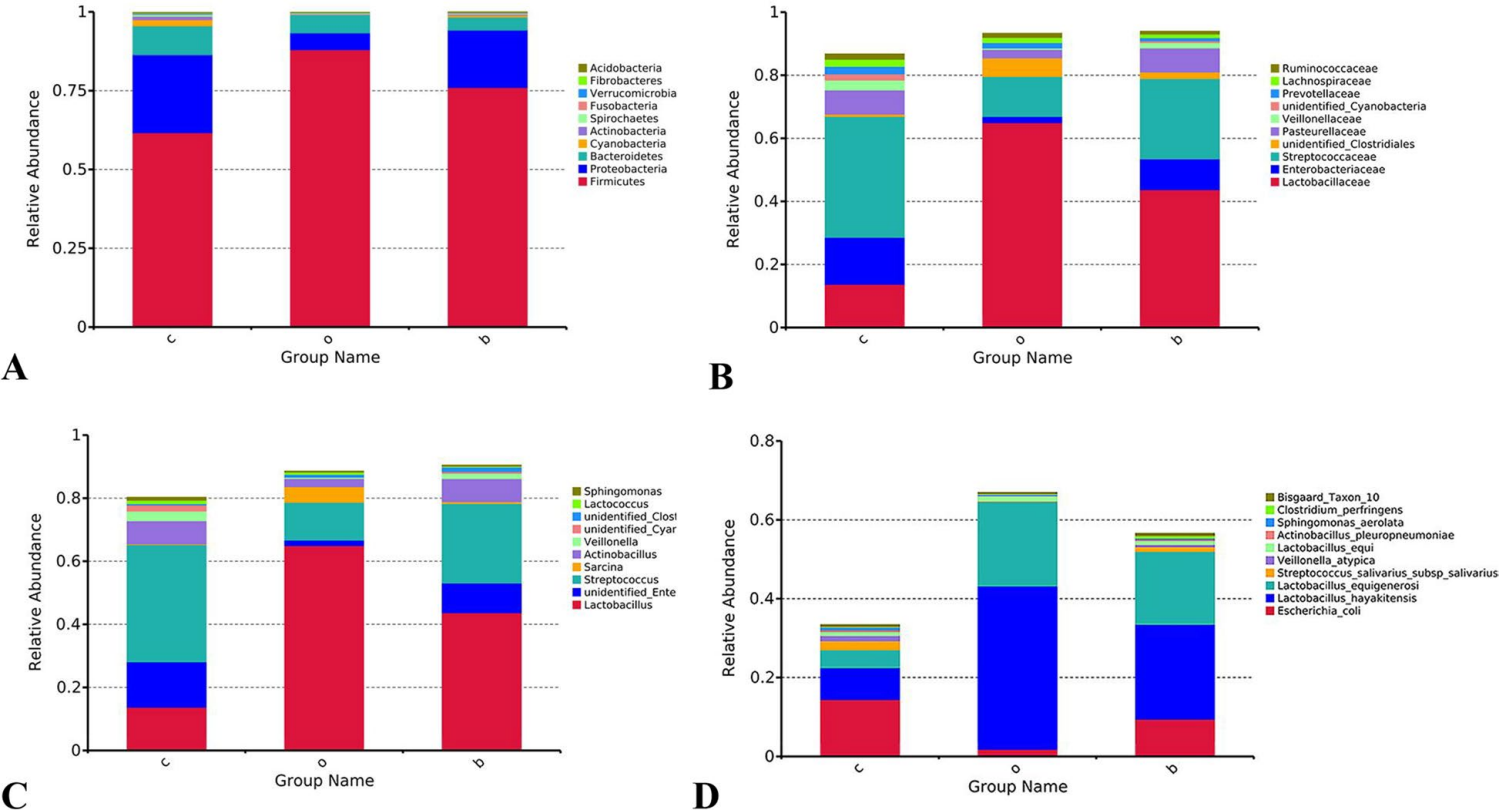
Effect of different grain sources on the relative abundance of bacteria in the stomach content of weaned foals at phylum **A**, family **B**, genus **C**, and species **D** levels. The vertical coordinates of the figure indicate the relative abundance of bacterial species and the horizontal coordinates indicate the groups, with c indicating the corn group, o indicating the oats group, and b indicating the barley group. The species names indicated by letters in the figure are shown in the legend on the right. “**A**” indicates histograms of the relative abundance of the top 10 bacterial phyla in the stomach content of foals. “**B**” indicates histograms of the relative abundance of the top 10 bacterial families in the stomach content of foals. “**C**” indicates histograms of the relative abundance of the top 10 bacterial genera in the stomach content of foals. “**D**” indicates histograms of the relative abundance of the top 10 bacterial species in the stomach content of foals

The relative abundances of bacteria in the stomach content at the phylum (A), family (B), genus (C), and species (D) levels are presented in Fig. 3. High-throughput sequencing demonstrated Firmicutes, Proteobacteria, Bacteroidetes, Cyanobacteria, Actinobacteria, Spirochaetes, Fusobacteria, Verrucomicrobia, Fibrobacteres, and Acidobacteria as the top 10 bacteria at the phylum level in the stomach content of foals, as evident in Fig. 3-A. The abundance of Firmicutes differed significantly between the oats and corn groups (88.03 and 61.69%, respectively, *P* < 0.05). The abundance of Cyanobacteria was significantly higher in the corn group than in the oats group (1.93 and 0.02%, respectively, *P* < 0.05). The abundance of Actinobacteria was significantly higher in the corn group (1.12%) than in the oats group (0.27%) and the barley group (0.33%) (*P* < 0.01). Moreover, the abundance of Fibrobacteres was significantly higher in the corn group than in the oats and barley groups (0.1, 0.02, and 0.02%, respectively, *P* < 0.05).

At the family level (Fig. 3-B), Lactobacillaceae, Enterobacteriaceae, Streptococcaceae, Unidentified_Clostridiales, Pasteurellaceae, Veillonellaceae, Unidentified_Cyanobacteria, Prevotellaceae, Lachnospiraceae, and Ruminococcaceae comprised the top 10 bacteria. Among them, Lactobacillaceae, Streptococcaceae, Unidentified_Clostridiales, Veillonellaceae, Lachnospiraceae, Ruminococcaceae belong to the Firmicutes. Enterobacteriaceae, Pasteurellaceae belong to the Proteobacteria. Unidentified_Cyanobacteria belong to the Cyanobacteria, Prevotellaceae belong to the Bacteroidetes. A highly significant (64.94 and 13.71%, respectively, *P* < 0.01) difference was observed in the abundance of Lactobacillaceae between the oats and corn groups. The abundance of Streptococcaceae was significantly higher in the corn group than in the oats group (38.31 and 12.68%, respectively, *P* < 0.05). The abundance of Unidentified_Clostridiales was significantly higher in the oats group (5.90%) than in the corn group (0.71%) (*P* < 0.05). The abundance of Prevotellaceae was significantly higher in the corn group than in the barley group (2.42 and 0.99%, respectively, *P* < 0.05).

At the genus level (Fig. 3-C), *Lactobacillus* (belongs to the Lactobacillaceae, Firmicutes), *unidentified_Enterobacteriaceae* (belongs to the Enterobacteriaceae, Proteobacteria), *Streptococcus* (belongs to the Streptococcaceae, Firmicutes), *Sarcina* (belongs to the Unidentified_Clostridiales, Firmicutes), *Actinobacillus* (belongs to the Pasteurellaceae, Proteobacteria), *Veillonella* (belongs to the Veillonellaceae, Firmicutes), *Unidentified_Cyanobacteria* (belongs to the Unidentified_Cyanobacteria, Cyanobacteria), *Unidentified_Clostridiales* (belongs to the Unidentified_Clostridiales, Firmicutes), *Lactococcus* (belongs to the Streptococcaceae, Firmicutes)*, and Sphingomonas* (belongs to the Sphingomonadaceae, Proteobacteria) were the top 10 bacteria. A highly significant difference was observed in the abundance of *Lactobacillus* between the oats and corn groups (64.95 and 13.71%, respectively, *P* < 0.01). On the other hand, the abundance of *Streptococcus* was significantly higher in the corn group than in the oats group (37.17 and 11.92%, respectively, *P* < 0.05). The abundance of *Unidentified_Cyanobacteria* was significantly higher in the corn group (1.93%) than in the oats group (0.03%) (*P* < 0.05). The abundance of *Unidentified_Clostridiales* was significantly higher in the barley group than in the corn groups (1.41 and 0.41%, respectively, *P* < 0.05). The abundance of *Lactococcus* was significantly higher in the corn group than in the barley group (1.10 and 0.28%, respectively, *P* < 0.05). *Sphingomonas* exhibited a significantly higher abundance in the corn group than in the oats group (0.86 and 0.25%, respectively, *P* < 0.05).

At the species level (Fig. 3-D), *Escherichia_coli* (belongs to the *Unidentified_Enterobacteriaceae*, Enterobacteriaceae, Proteobacteria), *Lactobacillus_hayakitensis* (belongs to the *Lactobacillus*, Lactobacillaceae, Firmicutes), *Lactobacillus_equigenerosi* (belongs to the *Lactobacillus*, Lactobacillaceae, Firmicutes), *Streptococcus_salivarius_subsp_salivarius* (belongs to the *Streptococcus*, Streptococcaceae, Firmicutes), *Veillonella_atypica* (belongs to the *Veillonella*, Veillonellaceae, Firmicutes), *Lactobacillus_equi* (belongs to the *Lactobacillus*, Lactobacillaceae, Firmicutes), *Actinobacillus_pleuropneumoniae* (belongs to the *Actinobacillus*, Pasteurellaceae, Proteobacteria), *Sphingomonas_aerolata* (belongs to the *Sphingomonas*, Sphingomonadaceae, Proteobacteria), *Clostridium_perfringens* (belongs to the *Unidentified_Clostridiales*, Unidentified_Clostridiales, Firmicutes)*, and Bisgaard_Taxon_10* (belongs to the Pasteurellaceae, Proteobacteria) constituted the 10 top bacteria. A highly significant difference was observed in the abundance of *Lactobacillus_hayakitensis* between the oats and corn groups (41.42 and 8.09%, respectively *P* < 0.01), while the abundance of *Lactobacillus_equigenerosi* was significantly higher in the oats (21.43%) and barley groups (18.48%) than in the corn group (4.50%) (*P* < 0.05). The abundance of *Clostridium_perfringens* in the barley group (0.63%) was significantly higher (*P* < 0.05) than that in the corn (0.11%) and oats (0.24%) groups.

### Prediction of function

**Fig. 4:**
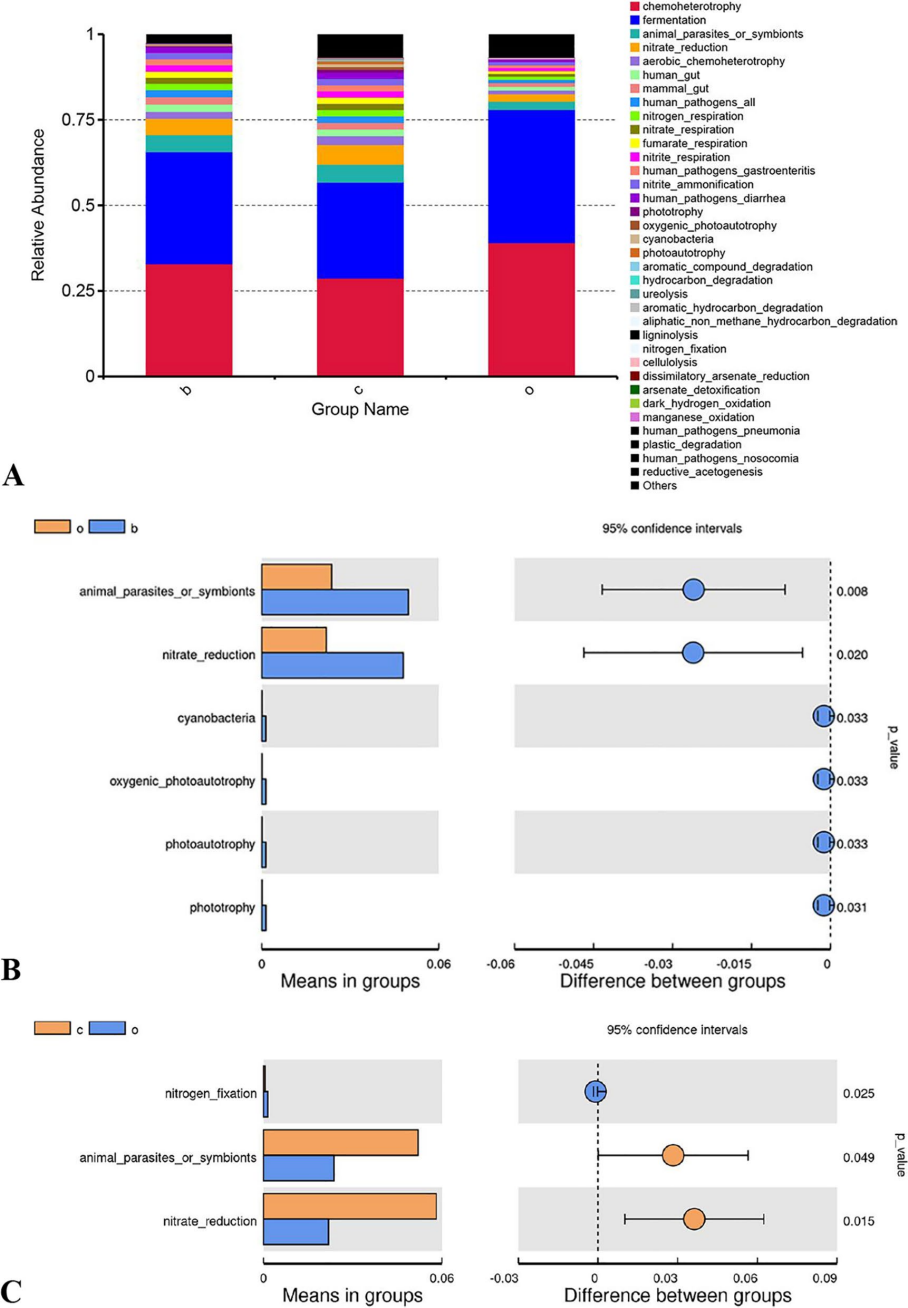
Effect of different sources of grains on functional prediction of bacteria in the stomach content of the weaned foals. “**A**” shows the histogram depicting the relative abundance of the first 35 functional bacteria predicted in the stomach content of the foals fed with three different diets. The vertical coordinates of the figure indicate the relative abundance of the predicted functions and the horizontal coordinates indicate the grouping, with c indicating the corn group, o indicating the oats group, and b indicating the barley group. The names of the functions indicated by letters in the figure are shown in the legend on the right. “**B**” compares the first 35 functional bacteria that were significantly different between the oats and barley groups by using *t-*test. The groupings indicated by the letters in the figure are shown in the legend at the top left. Orange and o indicate the oats group and blue and b indicate the barley group. “**C**” shows that the corn and oats groups were significantly different in the top 35 functional bacteria predicted using *t*-test. The groupings indicated by the letters in the figure are shown in the legend at the top left. Orange and c indicate the corn group and blue and o indicate the oats group

This study used the Tax4Fun [24] software for predicting the function of the stomach content samples. The functional analysis of the species information, performed using the FAPROTAX software, (Fig. 4-A) revealed a total of 35 functions. Feeding different grains to the horses in the different groups had some effects on the bacterial function in the stomach of the horses (Fig. 4-B and Fig. 4-C). Compared with the oats group, the barley group exhibited significant animal_parasites_or_symbionts (*P* = 0.008), nitrate_reduction (*P* = 0.020), cyanobacteria (*P* = 0.033), oxygenic_photoautotrophy (*P* = 0.033), photoautotrophy (*P* = 0.033), and phototrophy (*P* = 0.031). The oats group had significant nitrogen_fixation compared with the corn group (*P* = 0.025). On the contrary, the corn group had significant animal_parasites_or_symbionts (*P* = 0.049) and nitrate_reduction (*P* = 0.015) compared with the oats group.

### Linear discriminant analysis of effect size

**Fig. 5:**
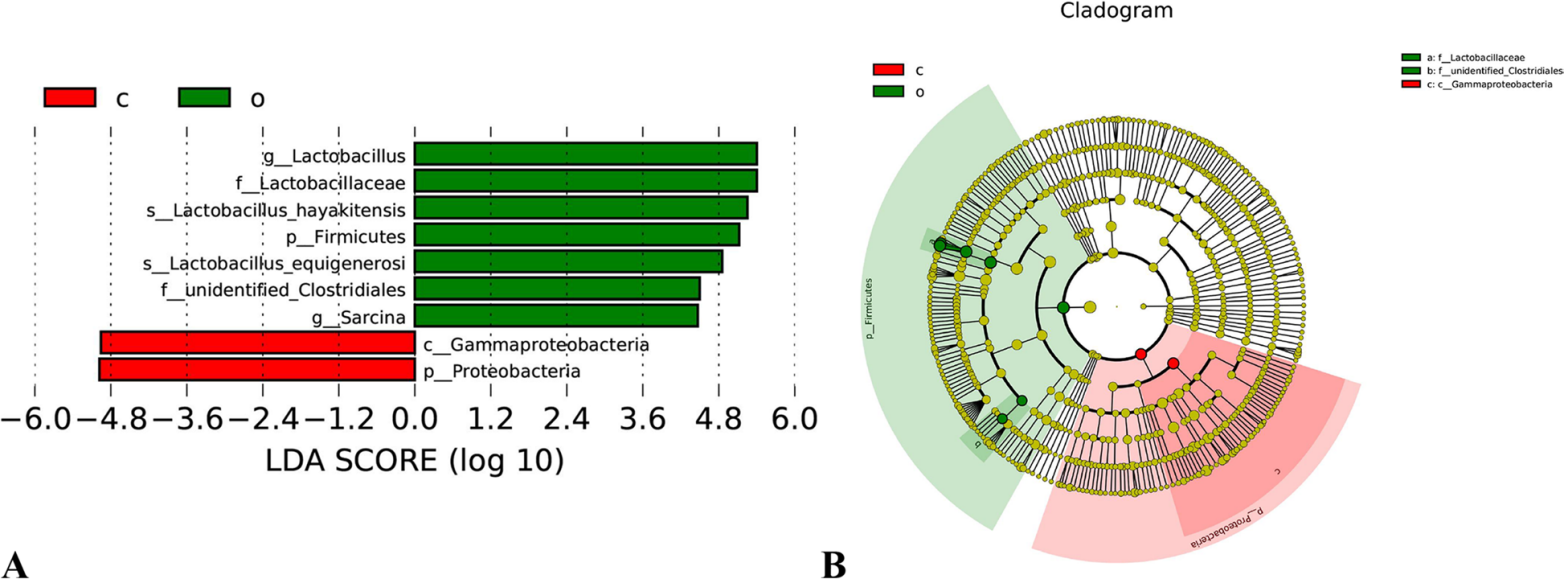
LEfSe analysis of statistically different biomarkers in stomach content. The non-parametric factorial Kruskal–Wallis Sum-Rank test (non-parametric Kruskal–Wallis and rank test) was used to detect the species with significant difference in bacterial abundance in the stomach content of foals from different grain sources, and then the difference between groups was determined using the Wilcoxon rank sum test. Finally, LDA was used to reduce and evaluate the impact size of significantly different species (i.e., LDA score). A Histogram of LDA value distribution, showing the biomarkers with statistical differences between groups with an LDA score greater than the set value of 4. The length of the bar chart represents the influence size of different species (i.e., LDA score). The groupings indicated by the letters of the alphabet are shown in the legend at the top left, Green and o represents species with significant abundance difference in the oats group, and red and c represents species with significant abundance difference in the corn group. B Evolutionary branching diagram obtained through the LEfSe analysis. In the figure, circles radiating from the inside to the outside represent taxonomic levels from phylum to genus (or species). Each small circle at different taxonomic levels represents a taxon at that level, and the diameter of the small circle is proportional to the relative abundance. The groupings indicated by the letters of the alphabet are shown in the legend at the top left, Green and o represents species with significant abundance difference in the oats group, and red and c represents species with significant abundance difference in the corn group

The LEfSe (LDA Effect Size) [25] was used to identify high-dimensional biomarkers for comparing the groups. Fig. 5 shows the characteristics of the different abundances and associated categories of the results. In this study (Fig. 5-A), different bacterial species were observed in the corn and oats groups. The bacteria that differed at the phylum level in the corn group were Proteobacteria and those that differing at the class level were Gammaproteobacteria. The study identified 7 species of bacteria that differed at each taxonomic level in the oats group; Firmicutes at the phylum level, Lactobacillaceae and Unidentified_Clostridiales at the family level, *Lactobacillus* and *Sarcina* at the genus level, and *Lactobacillus_hayakitensis* and *Lactobacillus_equigenerosi* at the species level. Fig. 5-B shows the evolutionary branching diagram, where the circles radiating from the inner to the outer represent the taxonomic levels from phylum to genus (or species). Each small circle at a different taxonomic level represents a taxon at that level, and the diameter of the small circles is proportional to the relative abundance size. Coloring principle: species without significant differences are uniformly colored yellow, and differential species biomarker follows the group for coloring. The red nodes indicate microbial taxa that play an important role in the corn group, being the phylum Proteobacteria and class Gammaproteobacteria. Green nodes indicate microbial taxa that play an important role in the oats group, mainly Lactobacillaceae and microbial strains of the Unidentified_Clostridiales family in the phylum Firmicutes. If a group is missing in the figure, it indicates that there are no significantly different species in this group and therefore the group is missing.

### The pH, glucose concentration, and enzyme viability analyses

Glucose levels and enzyme viability reflect starch digestion in the stomach of the horses fed with different diets. In this study (Table 4), pH, glucose concentration, and maltase, β-amylase, and amylase viabilities were not statistically different between the groups. However, the glucose production efficiency was higher in the corn group than in the oats and barley groups, and the glucose concentration in the stomach content of the corn group was 24.41% (*P* > 0.05) and 25.00% (*P* > 0.05) higher than that in the oats and barley groups. The maltase, β-amylase, and AMS amylase activities were higher in the corn group than in the oats and barley groups, which indicated that the foals had a higher level of digestion of corn starch in the stomach.

**Table 4:** Effect of different sources of grains on the pH, glucose concentration, and starch-digesting enzymes in the weaned foals stomach

	Corn group	Oats group	Barley group	SEM	*P* Value
pH	3.09	3.00	4.04	1.04	0.7443
Glucose mmol/gprot	7.85	6.31	6.28	1.77	0.7824
Maltase U/mgprot	173.96	165.43	136.10	20.90	0.4542
β-amylase U/mgprot	67.94	51.52	32.72	11.72	0.1856
Amylase U/mgprot	8.34	6.10	4.25	1.75	0.3251

## Discussion

Bacterial microbiota is critical for the health, growth, and absorption of digested products in animals. The equine gastrointestinal tract harbors several microbial species including bacteria, archaea, and eukarya (protozoa and fungi) [26, 27]. The gastrointestinal microbiota composition in humans and animals is affected by the foods ingested. Recent studies have indicated that the microbial communities and their functions are associated with the feed type [28]. However, the bacterial microbiota continue to develop throughout adolescence. Differences in the community structure are determined on the basis of the number and type of species as well as their relative abundances. The gastrointestinal tract microbiota is shaped by major events such as the initial colonization and stabilization of the microbiota, weaning, and switch to solid food [29]. Several studies have reported that the mature gut microbiota is permanently established when the solid food is converted and during weaning [30, 31]. To ensure a beneficial and lasting impact of the dietary source on the microbiota in weaning foals, the microbial communities should be identified, which constitutes the microbiota stabilized in the gastrointestinal tract of the weaning foals.

A deeper, more visual, and accurate picture of gastrointestinal health and growth is obtained through changes in the stability and diversity of the gut microbial community by using Illumina high-throughput sequencing, making this one of the preferred methods for studying complex gut microbial ecosystems and is widely used in microbiological research in recent years [32–35]. Sequencing the accurate 16S ribosomal DNA (rDNA) gene region is essential for determining the utility of microbial genomics for species-level assignments [36]. The present study reported each taxonomic level (phylum, family, genus, and species) based on the V3–V4 regions. Proudman et al. [37] sequenced 14 fecal samples from 8 thoroughbred geldings, obtaining a total of 488,213 valid sequences (average OTUs) 1200–3000). Yatsunenk et al. [38] detected more than 97% of microbial bacteria with over 2000 OTUs in human feces through high-throughput sequencing. Digestion of starch in cereals varies depending on several factors, with the most crucial factors being the level of intake, botanical origin, genotype, and feed processing of cereal grains. Meanwhile, the starch in cereal grains is primarily composed of amylose and amylopectin molecules arranged around a central helium and affects the internal ecology of the gastrointestinal tract. Bacteria and the gastrointestinal tract are interdependent and mutually beneficial. The type of diet not only affects the digestion and absorption of nutrients by animals but also influences the composition, structure, and diversity of gastrointestinal bacteria [11]. The present study detected an abundant number of OTUs in the stomach content of foals, with 979 OTUs in common and 592 unique to the corn group, 332 to the oats group, and 220 to the barley group. The corn group could significantly increase the ACE (823.04), Chao1 (805.19), and Shannon (4.29) indices of bacteria in the stomach of foals compared with the barley and oat groups. This difference was mainly related to the molecular structure of the constituent corn starch, and its content of amylose and amylopectin. The different content and ratio of amylose and amylopectin lead to different rates of starch digestion in the stomach, thus affecting the type and concentration of stomach nutrients, as well as the microbial ecology in the stomach, with implications for bacterial species and diversity [39–41].

Microbial composition of the gastrointestinal tract is influenced by the source, processing, and addition of grain starch in the diet [8, 42, 43]. Under low-grain dietary conditions, corn and wheat increased the abundance and number of starch-degrading bacteria in the gastrointestinal tract, while oats had no effect on these bacteria [43, 44]. Both oats and corn increased the abundance and number of starch-degrading bacteria when high-starch diets are consumed, and the abundance and number of starch-degrading bacteria in the equine gastrointestinal tract were more influenced by the amount of corn fed. These results were also observed in a study on in vitro horse manure fermentation [45]. Thus, the differences in bacterial composition and abundance in the stomach of foals were to some extent strongly influenced by the source of dietary grain starch. In our results, the top 10 bacteria at the phylum level were Firmicutes, Proteobacteria, Bacteroidetes, Cyanobacteria, Actinobacteria, Spirochaetes, Fusobacteria, Verrucomicrobia, Fibrobacteres, and Acidobacteria, which are similar to the results of studies on the human, pigs, and horse genera [46–50]. However, large differences in bacterial abundances were noted because of the different grains in the diets. The abundance of Cyanobacteria, Actinobacteria, and Fibrobacteres was significantly or highly significantly higher in the corn group than in the oat and barley groups. The abundance of Firmicutes was significantly higher in the oats group than in the other two groups. Studies have shown that increasing the diet starch content affects fiber digestibility [51]. Fiber digestibility decreases significantly when roughage is replaced with more than 60% oats [52, 53]. Similarly, replacing hay with barley decreases fiber digestibility in the horse’s large intestine. In a similar study, Medina et al. [54] reported that replacing alfalfa with barley significantly reduced the number of equine cecum fiber-degrading bacteria. In those studies, the increase in starch intake was confounded with a decrease in fiber intake. In our study, foals were given an equivalent starch diet, and the abundance of Fibrobacteres in the foal stomach in the corn group was significantly higher, probably related to the extent of enzymatic digestion of maize in the stomach. Moreover, microbial degradation of fiber occurs mainly in the cecum and colon of foals, and whether fiber digestion in the stomach is improved needs to be determined through studies such as those examining the extent of digestion of fiber-based feeds in the stomach.

The horse’s stomach is enriched in mucosal microflora, with *Lactobacillus* (belongs to the Lactobacillaceae, Firmicutes), *Streptococcus* (belongs to the Streptococcaceae, Firmicutes), and *Sarcina* (belongs to the Clostridiaceae, Firmicutes) bacteria being the most abundant genera [55]. This study identified Firmicutes, Bacteroidetes, and Proteobacteria as the most abundant microbiota at the phylum level, Lactobacillaceae and Streptococcaceae as the most abundant microbiota at the family level, and *Lactobacillus*, *Streptococcus,* and *Sarcina* bacteria as the most abundant microbiota at the genus level, consistent with the results of Pei et al. [55]. Microbial activity in the stomach cannot be ignored since it might be involved in the digestion of dietary starches. Varloud et al. [56] demonstrated that a significant amount of starch, approximately 41–76% disappears from the stomach of horses. Although the potential fermentation of dietary starch might alter its energetic dominance, the early disappearance of starch has not been measured in live horses or its relationship with the gastric microbiota has not been demonstrated.

The abundance of lactic acid bacteria and streptococcus is affected by dietary grain intake [47, 53, 57]. In the fecal study in foals, these genera led to gastrointestinal disturbances and laminitis [57–59]. Fernandes et al. [47] found that *Lactobacillus* was present at a 1% relative abundance in the fecal microbiomes, whereas no relative abundances of *Streptococcus* were observed in either group of horses, not even 1%, although a higher abundance of these genera was noted in the proximal regions of the hindgut than in the feces. Differences were noted in the samples obtained from horses fed with different combinations of the forage and grains as well as in the samples isolated from the cecum, colon, and feces [34, 52, 60, 61]. In our study, Firmicutes was the most abundant phylum in each of the three groups, and Lactobacillaceae and Streptococcaceae belonging to Firmicutes were the two most abundant bacteria at the family level. Significant or highly significant differences were noted in the abundance among the three groups, with Lactobacillaceae being the most abundant in the oats group (64.94%), followed by that in the corn (13.71%) and barley (43.69%) groups. The abundance of Streptococcaceae was significantly higher in the corn group (38.31%) than in the oats group (12.68%). At the genus level, *Lactobacillus* and *Streptococcus* were the more abundant genera in the three groups, with significant differences observed among the groups. Significant differences were observed between *Lactobacillus_hayakitensis* and *Lactobacillus_equigenerosi* at the species level. This suggested that cereal species had a significant effect on the bacterial composition of the stomach content, particularly on *Lactobacillus* and *Streptococcus*. Moreover, the supplemental feeding of oats and corn had the greatest effect on *Streptococcus*. However, whether stomach microbial populations shift with the variation in the types of grain requires further investigation. Furthermore, this study detected the number of unclassified bacterial families (Unidentified_Clostridiales, Unidentified_Cyanobacteria) and genera (*Unidentified_Enterobacteriaceae*, *Unidentified_Cyanobacteria*, *Unidentified_Clostridiales*) demonstrating the paucity of knowledge on the microbiota composition. Therefore, additional cultivation and non-cultivation-based studies in various horse populations are required for evaluating the abundance and occurrence of the unclassified organisms and for understanding their functional role in microbial fermentation in the hindgut.

The genus *Lactobacillus* includes a large number of species of lactic acid bacteria [62]. *Lactobacilli* are important members of healthy gastrointestinal tracts of mammals and some of them are frequently administered as probiotics for their beneficial roles in mammalian. The diversity of lactobacilli in the structure of the horse gastrointestinal tract and faeces has been investigated using culturing and molecular profiling methods, respectively [63, 64]. The species *Lactobacillus_equi*, *Lactobacillus_hayakitensis* and *Lactobacillus_equigenerosi* were first isolated from the horse gastrointestinal tract or faeces [65–67]. *Lactobacillus_hayakitensis* and *Lactobacillus_equigenerosi* are common species of lactic acid bacteria in the gastrointestinal tract of horse. In our study, we found three highly expressed abundance of lactic acid bacteria, they are *Lactobacillus_hayakitensis*, *Lactobacillus_equigenerosi*, and *Lactobacillus_equi* respectively. The expression levels of *Lactobacillus_hayakitensis* and *Lactobacillus_equigenerosi* in the stomach of oat fed foals were 41.42 and 21.43%, respectively. Studies have shown that *Lactobacillus_equigenerosi* is common in the horse intestine, alleviates the infection of Salmonella, and regulates intestinal flora [68]. In previous studies, some strains of the genus Ligilactobacillus have been shown to have probiotic effects, such as contributing to the regulation of intestinal flora [69], alleviation of colitis [70], and high antibacterial activity [71]. *Lactobacillus_equigenerosi* is a lactic acid bacterium that was first isolated in 2002 from the feces of horses in Japan [65]. It is the predominant bacterial species in healthy horse intestines [65]. One study of strains from horse feces samples showed that all samples contained *Lactobacillus_hayakitensis*, *Lactobacillus_equigenerosi*, and *Lactobacillus_equi* [72]. Grain is rich in arabinoxylan, xyloglucan, lignin, cellulose, hemicellulose, β-glucan and other dietary fibers, which can be used under the action of intestinal microorganisms and have a probiotic effect on improving the intestinal environment and flora composition [73]. Oat is rich in β-glucan. Studies have confirmed that as a probiotic, oat β-glucan can significantly increase the number of *Bifidobacterium* and *Lactobacillus* in the intestine and reduce the number of *Escherichia_coli* [74, 75]. In our study, the expressions of *Lactobacillus_hayakitensis* and *Lactobacillus_equigenerosi* in the stomach of foals in the oat group were significantly increased, and the abundance of *Escherichia_coli* in the oat group was only 1.79%, which was significantly lower than that in the corn and barley groups. This may be due to the abundant β-glucan composition in oat.

In our study, it was found that the abundance of *Clostridium_perfringens* in the stomach of foals in the barley group was extremely significantly elevated. *Clostridium_perfringens* is a class of gram-positive bacteria that produces a large amount of gas when decomposing sugar in animal tissues, and most strains have capsule formation during growth, so it is called *Clostridium_perfringens* [76]. Studies have found that the bacteria can secrete more than ten different toxins such as α, β, ε, ι, μ, λ, ν, γ, δ, К, η and θ [77] the first four have the greatest impact on the host, which is called the As lethal toxins [78], these two dozen toxins are all proteins and have certain biological activities, but their pathogenic mechanisms are obviously different and can cause completely different diseases. *Clostridium_perfringens* secreted by *Clostridium_perfringens* type A will secrete a large amount of enterotoxins when spores are formed [79].

Grains contain a large amount of non-starch polysaccharides (NSP), among which water-soluble non-starch polysaccharides are most likely to form gel-like viscous substances in the intestine, and the viscosity of intestinal contents increases, which affects the contact between chyme and intestinal villi, and then affects nutrients. The active diffusion and absorption of nutrients not only reduces the digestibility of nutrients, but also affects the balance of intestinal flora. Numerous studies have also confirmed that dietary non-starch polysaccharides increase the number of harmful intestinal bacteria. Annett et al. [80] studied the effect of wheat, barley and corn diets on the proliferation of *Clostridium_perfringens* type A in vitro and showed that bacterial proliferation in digested wheat and barley diets was significantly higher than that in digested corn diets. The incidence of necrotizing enteritis was higher in broilers fed the wheat and barley diets compared to the corn diets because the wheat and barley diets increased the proliferation of *Clostridium_perfringens*.

The contents of total non-starch polysaccharides in corn, oats and barley were 90 g/kg, 36 g/kg and 167 g/kg, respectively [81]. Therefore, the reason for the significant increase in the abundance of *Clostridium_perfringens* in the stomach of foals in the barley group in this study is related to the high content of non-starch polysaccharides in barley. In the process of raising foals, attention should be paid to the use of grains to reduce the intake of high non-starch polysaccharides and avoid the occurrence of intestinal diseases in foals.

Microorganisms in the gastrointestinal tract of animals are involved in multiple physiological functions of the host, including digestion and metabolism, immunity, growth, anti-inflammation, antioxidation, and anticancer. For example, *Clostridium* can inhibit stress-induced intestinal damage, promoting cancer cell apoptosis, and methanogenic bacteria can reduce the transit function of the small intestine [82]. Langille et al. [83] proposed a method that reverses the speculated function of microorganisms based on 16S equal marker genes. This method is more widely useful for predicting the function of intestinal microorganisms and for the in-depth study of the possible biological functions of microorganisms. This study was based on the existing 16S high-throughput sequencing and identified a total of 35 KEGG functional clusters through functional analysis of species information by using the FAPROTAX software. At the secondary functional classification level for microorganisms in the stomach content of foals, chemoheterotrophy, fermentation, animal_parasites_or_symbionts, nitrate_reduction, and aerobic_chemoheterotrophy were found to be the dominant functions. From these results, we inferred that the functional genes differ significantly between the groups, and this functional difference might have be related to the starch composition and structure in grains, besides having individual differences. The specific reasons for this however need further in-depth investigation using methods such as macrogenomics. Notably, although the functional gene could be predicted based on the widely used 16S sequences, the results need to be analyzed and applied cautiously since the method determines the gene function by comparing the sequences in reference databases, and relatively few studies have been conducted on the data available in the existing databases for horses. Moreover, this method can only obtain information on the OTUs through comparison with the database and might overlook the newly discovered species. Hence, further validation of the true functions of microorganisms in the stomach of foals requires more appropriate methods.

The main function of the stomach lies in digesting and inactivating ingested food and preventing microorganisms such as bacteria, viruses, fungi, and parasites from reaching the intestine [84]. This study used the LEfSe analysis and revealed 9 bacterial species to be significantly different between the groups fed with corn and oats: two in the corn group (Proteobacteria and Gammaproteobacteria) and seven in the oats group (Firmicutes, Lactobacillaceae, Unidentified_Clostridiales, *Lactobacillus*, *Sarcina*, *Lactobacillus_hayakitensis,* and *Lactobacillus_equigenerosi*). This result agree with the results of FAPROTAX functional prediction, which suggested that gastric microbial functions of the oats group mainly accounted for the maintenance of mammal_gut, human_gut, and nitrogen_respiration. On the one hand, *Lactobacillus* is a beneficial microorganism promoting the health of the gastrointestinal tract and host by competitively inhibiting infection and colonization by pathogenic bacteria [85]. On the other hand, β-glucan in oats might bind to the macrophages of the immune system, enhancing their activity and phagocytosis. Thus, it might enhance the body’s ability to resist diseases [86] and promote the healthy development of the gastrointestinal tract. The corn group mainly had gastric microbial functions of reductive_acetogenesis and aromatic_compound_degradation.

The extent of starch digestion in the horse’s stomach is controlled by major factors such as intakes and feed processing of starch. Both the mean feed retention time and the enzymatic activity in the stomach and foregut are influenced by physical and biochemical changes during the process. The apparent digestibility of cereal starch varies from 20 to 90% in the foregut depending on the process used. Starch undigested in the prececal segment undergoes microbial fermentation in the hindgut [87]. If the grain is highly fermentable and arrives at a high proportion in the fermentation chambers, the risk of inducing dysfunction in the hindgut is higher [88]. A similar impact is expected in the hindgut as well as the stomach of horses where numerous starch-utilizing bacteria exist. Further investigations are required to identify the process permitting the highest prececal digestibility and decreasing the hindgut fermentability of starch. The efficiency of grain digestion in the stomach and small intestine can be further improved if dietary grains used can be treated using the steam flaked technique. Differences in digesting different grains in the stomach can serve as indicators, including gastric pH, glucose concentration, and starch-digesting enzyme activity. According the study results, corn was better digested in the stomach of the foals. The increase in the glucose concentration was not exclusively accountable to the enzymatic digestion of dietary grains. Therefore, the extent to which the cereal starch is digested in the stomach of the genus requires to be determined through further in-depth studies conducted using tests identifying the animal’s ability to digest nutrients.

## Conclusions

This study established that different grains have no significant effect on the microbial diversity of the stomach content of the foal. However, the diversity in food grains significantly affected the relative bacterial abundances. Feeding oats was particularly found to significantly increase the abundance of species belonging to phylum Firmicutes, Lactobacillaceae, *Lactobacillus,* and *Lactobacillus_hayakitensis*. However, the grains had no significant effect on the pH which lowest in the oats group (important for faster gastric emptying and reduced gastric acid secretion), glucose concentration, and enzyme viability of the stomach content of foals.

## Data Availability

All data generated or analysed during this study are included in this published article. The datasets generated during the current study are available in the NCBI Database, [accession number: PRJNA895340. https://www.ncbi.nlm.nih.gov/sra/PRJNA895340].

## Footnotes

1 http://ccb.jhu.edu/software/FLASH/

2 http://qiime.org/scripts/split_libraries_fastq.html

3 https://www.arb-silva.de/

4 http://www.drive5.com/usearch/manual/uchime_algo.html

5 http://drive5.com/uparse/

6 http://www.arb-silva.de/

7 http://www.drive5.com/muscle/
